# The solute-binding proteins DppA1–5 of *Pseudomonas aeruginosa* have distinct substrate profiles

**DOI:** 10.1038/s41598-025-27865-2

**Published:** 2025-12-23

**Authors:** Konstantin Plöchl, Thomas Böttcher

**Affiliations:** 1 Faculty of Chemistry, Institute of Biological Chemistry & Centre for Microbiology and Environmental Systems Science, University of Vienna, Vienna, Austria; 2 Vienna Doctoral School in Chemistry, University of Vienna, Vienna, Austria

**Keywords:** Peptides, Peptides, Metabolic pathways

## Abstract

*Pseudomonas aeruginosa* is an opportunistic pathogen with high antibiotic resistance that infects immunocompromised patients. Its survival in diverse environments relies on nutrient uptake systems such as the dipeptide permease (Dpp), an ATP-binding cassette transporter that imports di- and tripeptides. Unlike other bacteria, whose Dpp includes a single solute-binding protein that binds substrates in the periplasm, *P. aeruginosa* encodes five paralogs (DppA1–5), the functional significance of which remains unclear. Here, we systematically profile the ligand specificities of all five DppA proteins using Differential Scanning Fluorimetry with a library of 281 di- and tripeptides. We find that DppA1 and DppA3 preferentially bind dipeptides, whereas DppA2 and DppA4 favor tripeptides. DppA5 shows no detectable binding, suggesting a divergent function. Ligand binding is highly structure-sensitive and distinct across DppA paralogs. A comparative reanalysis of published nutrient utilization data for *dppA1–5* suggests that previous studies underestimated this functional specialization. In contrast, our findings indicate that *P. aeruginosa* encodes multiple DppA paralogs to expand Dpp substrate scope and enhance nutrient acquisition. This work provides a foundation for further exploration of the Dpp in bacterial signaling and its exploitation for drug delivery via Trojan Horse antimicrobials.

**Supplementary Information:**

The online version contains supplementary material available at 10.1038/s41598-025-27865-2.

## Introduction

Bacterial infections account for more than one in eight deaths globally^1^. The fifth-deadliest bacterial pathogen is *Pseudomonas aeruginosa*, responsible for 559,000 deaths and 18.9 million years of life lost in 2019^1^. This ubiquitous Gram-negative bacterium is found everywhere from the soil to sewages^2^. As an opportunistic human pathogen, *P. aeruginosa* preferentially infects immunocompromised individuals with chronic conditions such as cystic fibrosis or cancer and forms biofilms on medical implants and wounds, thus complicating treatment^3^. Biofilm formation, a highly impermeable outer membrane, and efflux pumps impart a high intrinsic antibiotic resistance^3^, prompting the World Health Organization to assign *P. aeruginosa* the highest priority for antimicrobial research^4^.

**Fig. 1:**
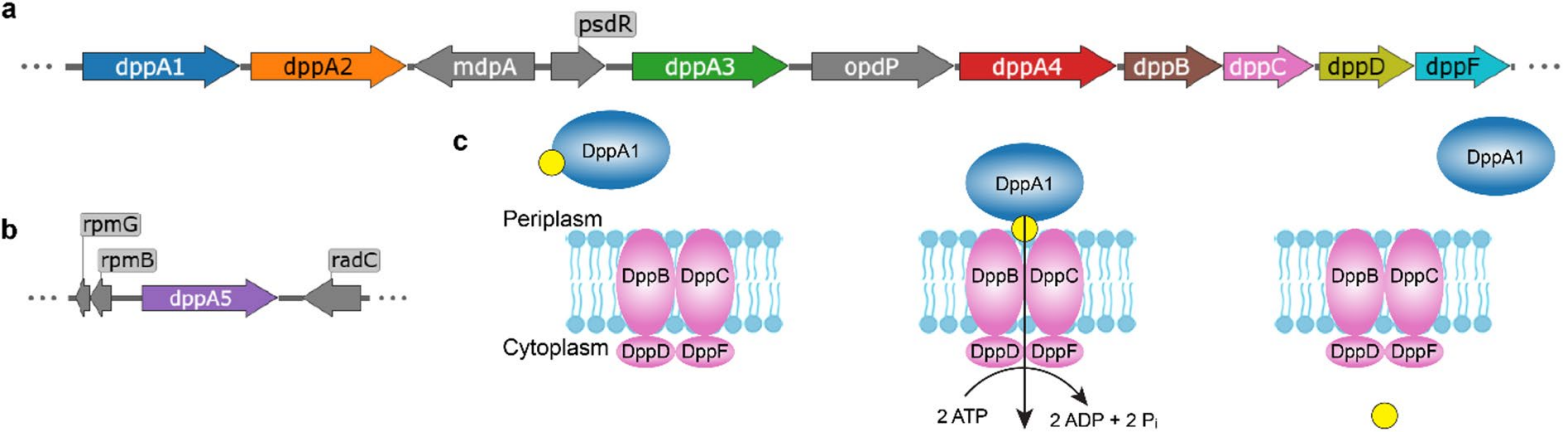
The dipeptide permease of *P. aeruginosa*. (**a**) The ABC transporter operon *dppBCDF* is located next to the SBPs *dppA1–4* on the *P. aeruginosa* PA14 genome. (**b**) The SBP *dppA5* is located distantly. (**c**) Schematic representation of the Dpp. A DppA protein binds its substrate (shown in yellow) in the periplasm and delivers it to the ABC transporter DppBCDF. This complex translocates the substrate across the bacterial inner membrane, powered by ATP hydrolysis.

The survival of *P. aeruginosa* in diverse and hostile environments is facilitated by its ability to utilize a broad spectrum of nutrient sources^2^. The uptake of nutrients is enabled by a plethora of transport systems, which constitute 10% of open reading frames in the *P. aeruginosa* genome^5^. Three operons encode peptide ATP-binding cassette (ABC) transporters, but only the dipeptide permease (Dpp) takes up unmodified peptides resulting from extracellular proteolysis^6–9^. Following entry into the periplasm through porins, di- and tripeptides bind to the solute-binding proteins (SBPs) DppA1–5, which deliver their substrates to the ABC transporter DppBCDF embedded in the cytoplasmic membrane (Fig. 1c). There, the SBPs release their cargo, and DppBCDF transports the peptides into the cytoplasm under ATP consumption. The *dpp* operon moreover encodes the outer-membrane porin OpdP, the metallopeptidase MdpA, and the transcriptional regulator PsdR^6,10^ (Fig. 1a). The gene *dppA5* is not found on the core *dpp* operon, but at a distant genomic location (Fig. 1b).

In addition to nutrient import, the Dpp also functions as a chemosensor. It is thought that ligand binding induces conformational changes in the SBPs, thereby modulating interactions with signaling proteins^11^. Mutational studies have demonstrated that *dppA1* is essential for biofilm formation and maintenance of Pf5 phage lysogeny^12^, and *dppA1* and *dppA2* are involved in tryptone chemotaxis^11^. Given that biofilm formation and motility are hallmarks of *P. aeruginosa* virulence, the Dpp plays a role beyond nutrient acquisition.

Whereas some bacterial Dpp systems include a single SBP, others comprise several^6^. *P. aeruginosa* encodes five DppA proteins, the functional significance of which remains unclear^13,14^. It has been hypothesized that these paralogs possess distinct ligand binding profiles, enabling the transport of a broader range of substrates^7^. Alternatively, they may be involved in separate signaling pathways. Previous investigations of substrate profiles using Differential Scanning Fluorimetry (DSF) were limited to DppA2 and DppA3 and did thus not permit a broader comparison^15^.

In this study, we systematically determined the ligand profiles of all five DppA proteins of *P. aeruginosa* by screening a library of 281 di- and tripeptides using DSF. Our findings reveal distinct substrate specificities for DppA1–5, enabling the extraction of ligand binding patterns. We further compare these results to previous genetic studies of *dppA1–5* through a reanalysis of published raw data^7^ and discuss discrepancies. Additionally, we computed protein structures of DppA1–5, which provide insights that corroborate our experimental findings.

## Results and discussion

### High-throughput ligand screening

**Fig. 2:**
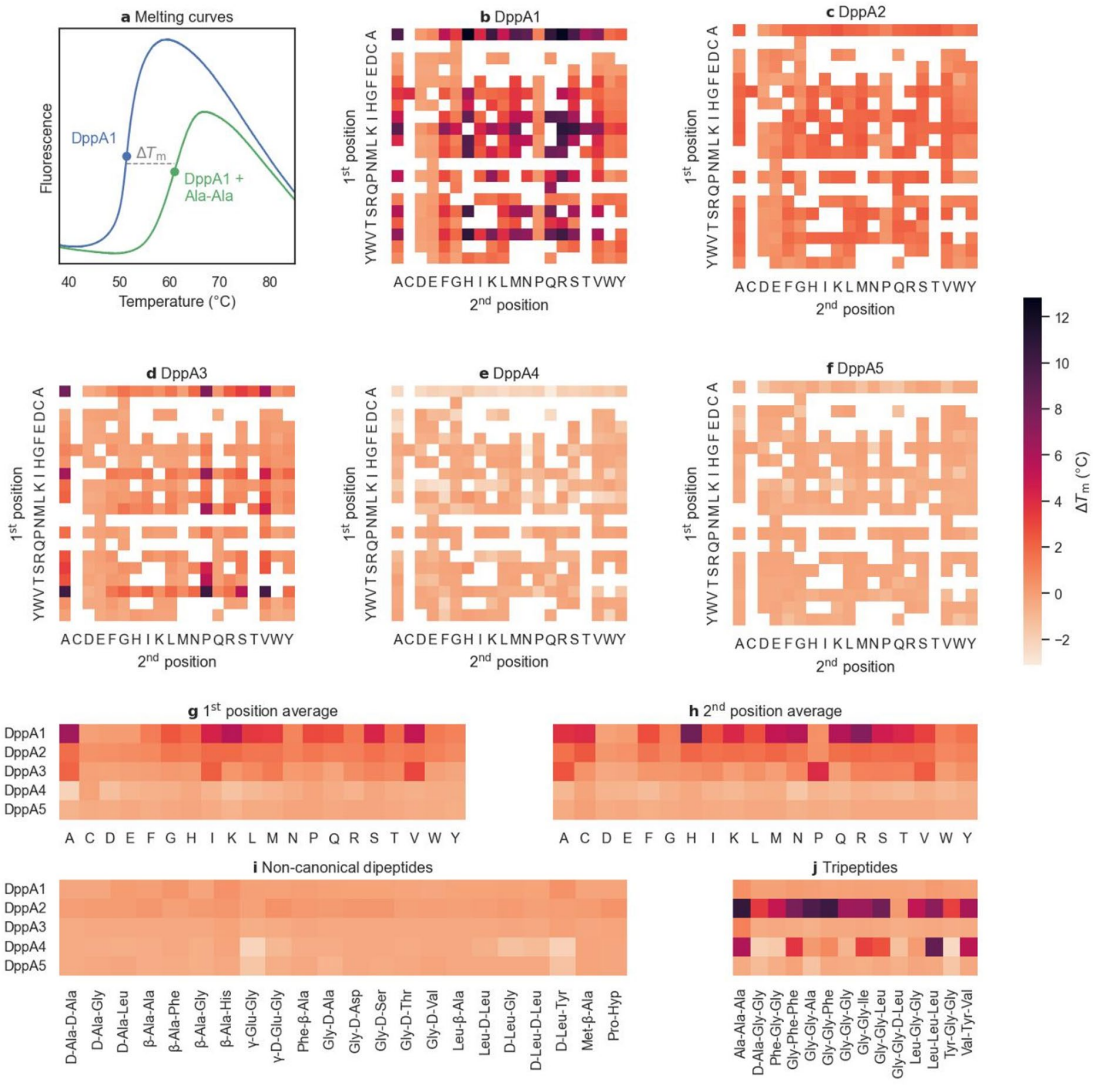
DSF of DppA1–5. (**a**) Melting curves of DppA1 in the presence and absence of the ligand Ala-Ala, with melting points indicated. (**b**−**f**) Thermal shifts of canonical dipeptides for DppA1–5. (**g**, **h**) Average Δ*T*_m_ for the 1st (**g**) and 2nd (**h**) position of canonical dipeptides. (**i**, **j**) Thermal shifts of non-canoncial dipeptides (**i**) and tripeptides (**j**). Hyp, 4-hydroxyproline.

Recombinant DppA1–5 were expressed in the periplasm of *Escherichia coli* and purified via StrepTag technology. Ligand binding was assessed using DSF, which detects protein unfolding by monitoring the fluorescence of a dye that selectively binds exposed hydrophobic regions. Ligand binding stabilizes the protein against thermal unfolding, and the resulting change in melting temperature (thermal shift, Δ*T*_m_) serves as a proxy for binding affinity (Fig. 2a)^16^. We recorded melting curves for DppA1–5 with a commercial library of 281 di- and tripeptides comprising 245 canonical dipeptides (i.e., containing only proteinogenic amino acids), 22 dipeptides containing non-canonical amino acids, and 14 tripeptides.

We validated the assay ex-post by eightfold replication of the strongest ligand (i.e., highest Δ*T*_m_) and the blank, yielding excellent *Z*-factors (≥ 0.8) for DppA1–4 but a low value (–1.12) for DppA5 (discussed below) (Table 1). Moreover, our results are in good accordance with data published^15^ for DppA2 (Pearson’s *R* = 0.98, *n* = 11 ligands) and DppA3 (*R* = 0.91, *n* = 53).

**Table 1:** Variations in DppA substrate preferences.

	DppA1	DppA2	DppA3	DppA4	DppA5
Mean Δ*T*_m_ (°C)	2.6	1.5	0.7	–0.7	–0.5
Max Δ*T*_m_ (°C)	12.8	10.6	10.3	8.8	–0.01^a^
Max Δ*T*_m_ ligand	Ala-His	Ala-Ala-Ala	Val-Pro	Leu-Leu-Leu	Gly-Cys
*Z*-factor	0.94	0.81	0.91	0.80	–1.12
*Δ*_aromatic_^b^	–43%	–13%	–76%	–4%	5%
*R*_hydrophobicity_^c^	–0.04	0.17	0.00	0.15	–0.04
*R*_charge_^d^	0.44	0.24	0.13	–0.01	–0.04
*Δ*_negative_^e^	–99%	–72%	–136%	31%	1%
*Δ*_neutral_^e^	3%	13%	32%	–13%	–1%
*Δ*_positive_^e^	89%	16%	–7%	25%	3%
RMSD^f^ (Å)	—	4.1	4.8	4.6	9.4

^a^No ligand exhibited a positive Δ*T*_m_ with DppA5. ^b^Relative change in Δ*T*_m_ of ligands containing at least one of Phe, Tyr, or Trp. ^c^Correlation coefficient between Wimley-White hydrophobicity of ligands (Methods) and Δ*T*_m_. ^d^Correlation coefficient between overall ligand charge at neutral pH and Δ*T*_m_. ^e^Relative change in Δ*T*_m_ of ligands with the specified overall charge at neutral pH. ^f^Root mean square deviation of backbone atom positions of superimposed DppA1–5 structures referenced to DppA1.

### DppA1–5 substrate profiles

The ligand profiles of DppA1–5 exhibited distinct characteristics. The range of thermal shifts varied considerably, with numerous instances of negative Δ*T*_m_, indicative of either protein destabilization or kinetic partitioning by a validated ligand to an unfolded state^17^. DppA1 and DppA3 strongly bound canonical dipeptides, whereas DppA2 showed weaker, and DppA4 and DppA5 no affinity for these ligands (Fig. 2b–f). Only DppA2 and DppA4 bound tripeptides (Fig. 2j). Isothermal titration calorimetry of DppA1 and DppA3 with the generic peptides Ala-Ala and Ala-Ala-Ala confirmed these proteins’ preference of dipeptides over tripeptides (Fig. S1). No protein bound dipeptides featuring non-canonical d-amino acids, β-amino acids, or γ-Glu linkages (Fig. 2i), supporting the notion that the primary function of the Dpp is uptake of digestion products of extracellular proteolysis^6^.

DppA5 was a notable exception, as no ligand exhibited a positive Δ*T*_m_ (Table 1). Di- and tripeptides are apparently not the endogenous ligands of this SBP. The negative *Z*-factor underscores that there was no significant difference detected between the best ligand and the blank measurement. The annotation of *PA14_70200* as *dppA5* was based on sequence homology with *dppA1–4*^7^; however, its genomic separation from the *dpp* operon and inability to bind di- and tripeptides suggest a different function.

In general, ligand binding was highly sensitive to small changes in ligand structure, as illustrated by DppA1’s high affinity for Ala-Ala (Δ*T*_m_ = 13.8 °C), which decreased significantly upon methyl group removal in the second position (Ala-Gly, 8.6 °C) and was abolished by removal in the first position (Gly-Ala, − 0.3 °C), migration of one amino group (β-Ala-Ala, 0.9 °C) or chiral inversion (d-Ala-d-Ala, − 0.3 °C).

Several DppA proteins showed preferences for canonical dipeptides containing distinct amino acids (Fig. 2g,h). DppA1 exhibited higher affinity for dipeptides with the basic amino acids His and Arg at the second position, whereas DppA3 preferred the hydrophobic amino acids Ala, Ile, and Val at the first position and Pro at the second position. All proteins disfavored acidic amino acids (Asp, Glu) in both positions.

Further physicochemical analysis revealed that canonical dipeptides containing aromatic residues yielded lower thermal shifts, although ligand hydrophobicity correlated only weakly with Δ*T*_m_ (Table 1). In contrast, ligand charge had a pronounced influence. DppA1–3 preferred neutral over negatively charged substrates, and DppA1 and DppA2 exhibited the highest thermal shifts for positively charged ligands (Table 1). This trend is further illustrated when ranking canonical dipeptides according to their maximum Δ*T*_m_ across DppA1–5 (Fig. S2). Notably, most neutral and positively charged dipeptides had a strong binding partner, whereas a majority of negatively charged dipeptides did not.

### Comparison with nutrient utilization assay

**Fig. 3:**
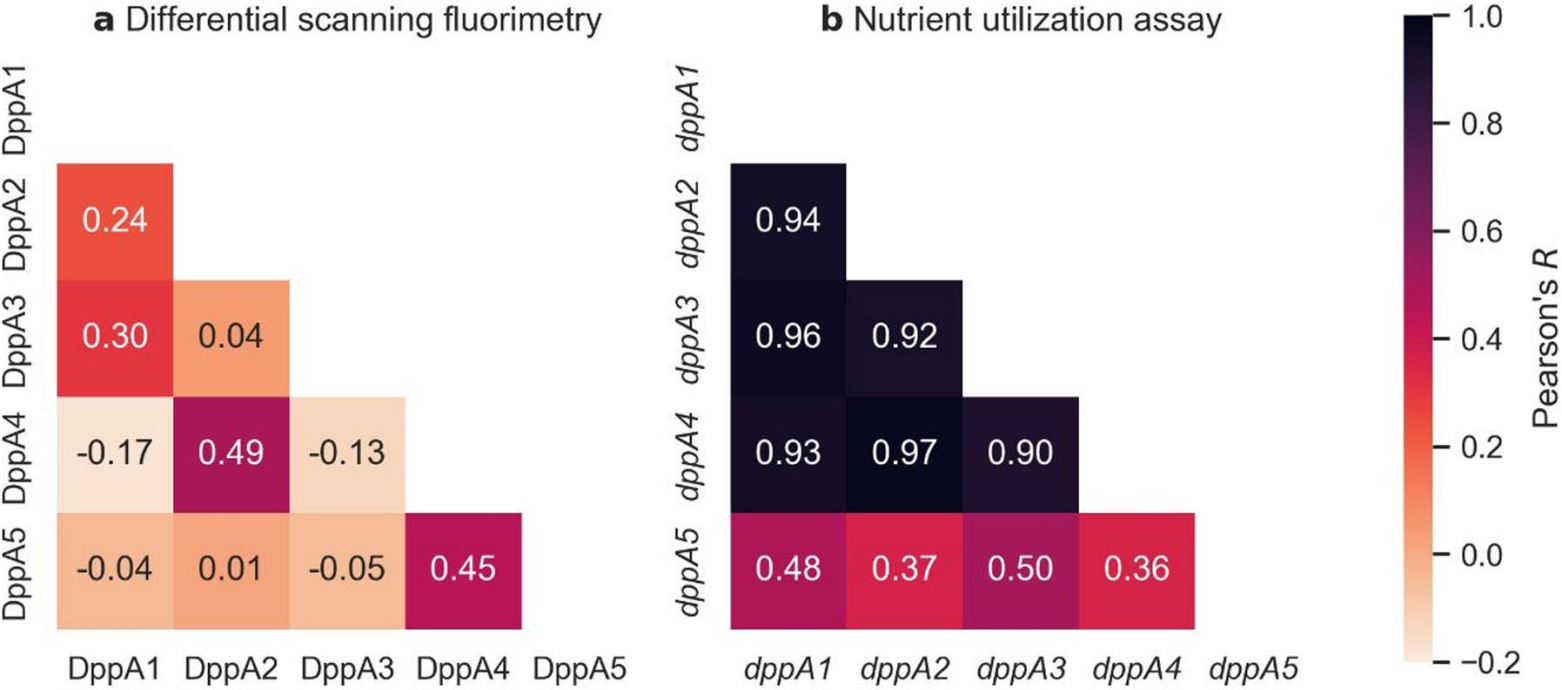
Correlations between DppA1–5 substrate profiles. Correlation coefficients of thermal shifts (**a**) and nutrient utilization (**b**) calculated from the data of Pletzer et al.^7^.

The distinct substrate specificities of DppA1–5, as determined by DSF, are reflected in the correlation coefficients of thermal shifts among the SBPs (Fig. 3a). The strongest correlations were observed between DppA1 and DppA3 (primarily dipeptide-binding SBPs), DppA2 and DppA4 (tripeptide-binding SBPs), and, unexpectedly, DppA4 and DppA5, both of which exhibited significant negative thermal shifts with dipeptides. No correlation exceeded 0.5, and several coefficients were negative, indicating largely orthogonal substrate profiles. These findings support the hypothesis that *P. aeruginosa* encodes five DppA paralogs to facilitate the uptake of a broader range of substrates than a single SBP could accommodate.

To further evaluate the relevance of distinct substrate profiles, we compared our findings to a previous study in which individual *dppA* genes were exogenously expressed in a *dppA1–5* deletion mutant to assess their respective contributions to nutrient utilization^7^. In this study, Pletzer et al. measured respiratory activity in minimal medium containing individual substances from the same commercial di- and tripeptide library used in our experiments, each serving as the sole nitrogen source^7^. Using their published raw data, we quantified di- and tripeptide utilization attributable to a single *dppA* gene as the increase in respiratory activity upon complementation of the *dppA1–5* mutant with that gene, using this as a proxy for substrate affinity to calculate correlations.

Strikingly, with the exception of *dppA5*, all correlation coefficients exceeded 0.9, indicating highly similar substrate utilization across *dppA1–4* (Fig. 3b). The authors of the study interpreted their results as evidence that multiple DppA proteins have similar substrate profiles, stating that their “data suggests that several SBPs have no clear preference for dipeptides with a specific side chain”^7^. However, we propose that this conclusion arises from a misinterpretation of the respiratory assay. Given the high substrate concentration used (4.5 mM according to the compound library manufacturer), it is likely that DppA binding sites were saturated, making substrate translocation via DppBCDF rather than SBP binding plausibly the rate-limiting step in uptake. Even though the peptides were growth-limiting as sole nitrogen sources, the assay setup cannot disentangle whether binding to DppA proteins or subsequent translocation and catabolism were limiting utilization. This effect could explain the high correlation in nutrient utilization across *dppA1–4*.

In contrast, the lower correlation coefficients for *dppA5* reinforce the conclusion that this SBP does not bind di- and tripeptides, even at high concentrations. Thus, the value of Pletzer et al.’s study^7^ lies in its detailed characterization of the translocation capacity of DppBCDF with respect to different di- and tripeptides, providing a substrate profile of the transporter rather than of individual SBPs. Our work complements these findings with direct in vitro measurements of ligand binding using purified DppA1–5.

### Structural analysis of DppA1–5

**Fig. 4:**
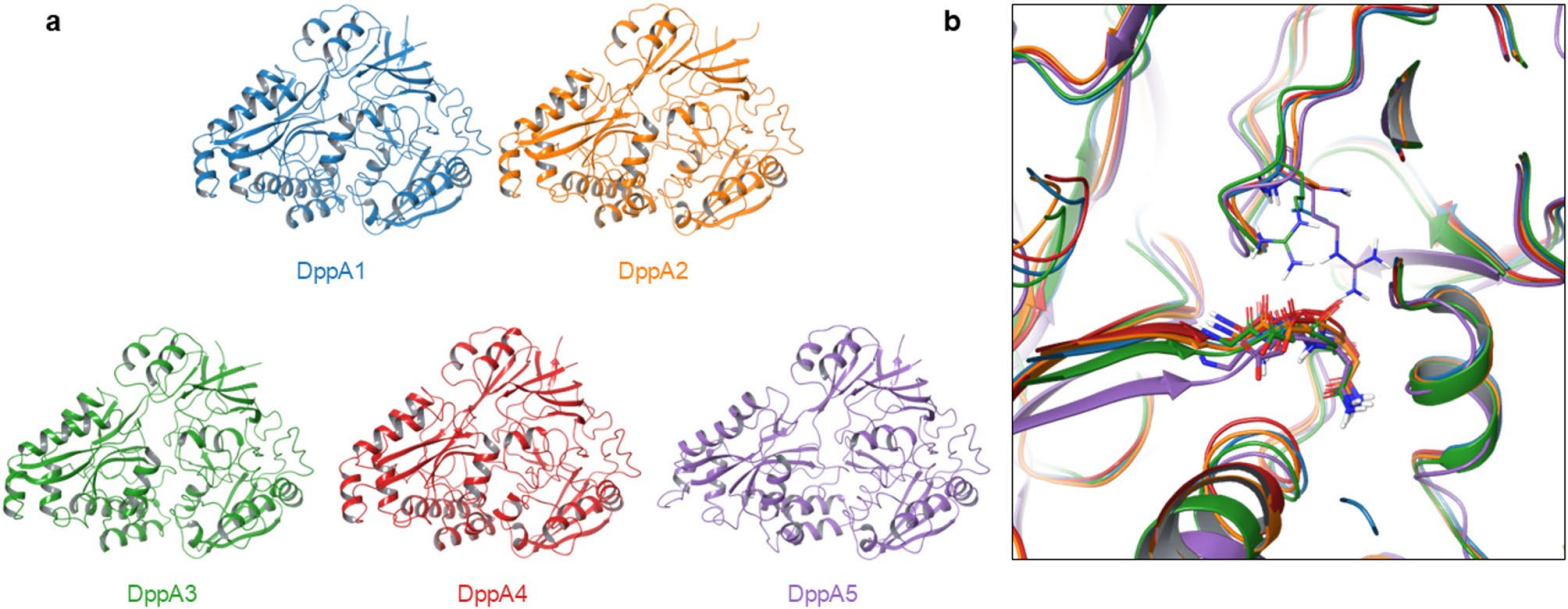
Predicted structures of DppA1–5. (**a**) Overall structures with the α-helix covering the binding pocket in the center. (**b**) Blowup of the binding pockets with the Gly-Asp-Asn motif and the covering residue highlighted.

To gain structural insights into the ligand-binding properties of DppA1–5, we computed their structures using AlphaFold2^18^. SBPs consist of two lobes connected by a hinge region, with the substrate binding to the interface of the lobes^19^. The predicted structures closely resemble the experimentally determined structure of *E. coli* DppA bound to Gly-Leu (PDB 1DPP)^20^ and can therefore be equally assigned to SBP cluster C^21,22^. They feature a central ligand pocket covered by an α-helix (Fig. 4a). A Gly-Asp-Asn motif within this pocket is conserved across *E. coli* DppA and *P. aeruginosa* DppA1–5 (Fig. 4b). In *E. coli*, the negatively charged Asp residue forms an ionic interaction with the N-terminus of the substrate; a feature that is likely conserved in *P. aeruginosa*. This interaction might also explain the lower thermal shifts observed for negatively charged ligands, suggesting that electrostatic repulsion weakens their binding (Table 1). The binding pocket is covered from the top by Leu498 in DppA1, Arg499 in DppA3, and Arg495 in DppA5. Ala499 in DppA2 is smaller; and Asn498 in DppA4 points away from the binding pocket (Fig. 4b). The absence of a bulky residue reaching into the binding pocket could explain the affinity of DppA2 and DppA4 for tripeptides. Moreover, the positively charged Arg499 in DppA3 could contribute to its decreased affinity for positively charged ligands (Table 1).

Among DppA1–5, DppA5 exhibited the greatest structural divergence, with the α-helix covering the binding pocket displaced (Fig. 4a). The root mean square deviation of backbone atom positions relative to DppA1 was < 5 Å for DppA2–4, but 9.4 Å for DppA5 (Table 1). These structural differences further support the conclusion that DppA5 does not share the same endogenous ligands as DppA1–4.

## Conclusion

The design of novel therapies to combat antimicrobial resistance requires a comprehensive understanding of bacterial physiology. In this regard, bacterial transport systems, such as the Dpp of *P. aeruginosa*, are particularly important due to their multifaceted roles. In addition to mediating nutrient uptake, which directly supports bacterial survival, these systems function as chemosensors integrated into signaling pathways that regulate virulence. For instance, DppA1 is involved in controlling Pf phage lysogeny^12^, which has been associated with worse outcomes in *P. aeruginosa* infections^23,24^. Moreover, the hijacking of bacterial transporters underpins the Trojan Horse strategy in antimicrobial design, wherein a known transporter substrate is conjugated to an antibiotic compound^25^. Notably, the Dpp of *P. aeruginosa* transports the toxic tripeptide phaseolotoxin produced by *Pseudomonas syringae*^7^, while the related nucleoside peptide permease (Npp) is essential for the uptake of peptidyl nucleoside antibiotics such as pacidamycin^8,26^.

In this study, we characterized the SBPs of the *P. aeruginosa* Dpp through in vitro measurements of ligand binding. Our results reveal distinct substrate specificities: DppA1 and DppA3 primarily bind dipeptides, whereas DppA2 and DppA4 favor tripeptides. These differences stand in contrast to the minimal variations reported previously using an indirect measurement method^7^. DppA5 did not share any ligands with DppA1–4, and its endogenous ligands remain unidentified. This observation, together with its distant genomic localization and predicted structural differences, suggests that DppA5 either possesses a highly distinct substrate scope or may not function as a component of the Dpp. It should be noted, however, that thermal shifts measured by DSF serve as a proxy for ligand affinity and do not yield direct thermodynamic binding parameters.

In summary, our findings support the hypothesis that the Dpp of *P. aeruginosa* comprises multiple SBPs to broaden the range of transported substrates. We anticipate that the detailed ligand profiles presented here will provide a valuable foundation for the development of Trojan Horse antimicrobials hijacking the Dpp and elucidation of its role in bacterial chemosensing.

## Materials and methods

### Protein expression and purification

The genes encoding *P. aeruginosa* PA14 DppA1–5 (UniProt IDs Q02GU5, Q02GU4, Q02GU1, Q02GT9, and Q02E45, respectively) were codon-optimized for *E. coli*, synthesized with a C-terminal TwinStrepTag (WSHPQFEKGGGSGGGSGGSAWSHPQFEK), and cloned into pET22b vectors for periplasmic expression (Genscript). The plasmids were transformed into *E. coli* BL21. Overnight cultures were diluted to OD = 0.05 with 1 L LB medium (Carl Roth X964.4) supplemented with 100 mg/L ampicillin (Carl Roth K029.5) and incubated at 37 °C with shaking at 180 rpm. At OD = 0.8, 100 µM IPTG (Carl Roth 2316.4) was added, and the cultures were shifted to 30 °C and incubated overnight. On the next day, the cells were harvested by centrifugation at 7000×*g* and 4 °C for 30 min. The supernatant was discarded, and the cells were resuspended in 15 mL chilled PBS (Carl Roth 0890.2) and lysed by sonication (Branson Digital Sonifier 250). The lysate was centrifuged at 80,000×*g* and 4 °C for 15 min. The supernatant was loaded onto a StrepTrap HP column (column volume 5 mL, Cytiva 28–9075−48) connected to an Äkta pure chromatograph (flow rate 5 mL/min, Cytiva 29018226) at 4 °C. After equilibration with 5 CV PBS, sample loading, and washing with 5 CV PBS, the target protein was eluted with a gradient of 0 to 2.5 mM desthiobiotin (BLD BD123554) over 7 CV, and the column was regenerated with 5 CV 0.5 M NaOH. The target fractions were concentrated by ultrafiltration (30 kDa cutoff, Millipore UFC9030) and stored with 20 vol% glycerol (Fisher BioReagents BP2291) at − 80 °C.

### Differential scanning fluorimetry

The compound library was composed of the PM6, PM7, and PM8 Phenotype MicroArray plates (Biolog 12181, 12182, and 12183, respectively). DSF reactions contained 10 µM protein, 250 µM peptide ligand, and 5× SYPRO Orange (Invitrogen S6650) in 10 µL PBS in white 96-well PCR plates (Brand 781364). The plates were incubated at 37 °C for 5 min and then heated to 85 °C over 1 h 26 min with excitation at 470 nm and detection at 514 nm (Roche Lightcycler 96). Melting temperatures (*T*_m_) were calculated as the temperature at the point of inflection of the melting curves, and thermal shifts (Δ*T*_m_) were calculated as the difference to the *T*_m_ of the protein without ligand.

### Assay validation

For each DppA protein, DSF was replicated eightfold as above with the ligand eliciting the highest Δ*T*_m_ and the blank (PBS). *Z*-factors were calculated as *Z* = 1–3 × (σ_ligand_ + σ_blank_)/|µ_ligand_ – µ_blank_|, where σ is the standard deviation and µ the mean of *T*_m_, respectively.

### Isothermal titration calorimetry

Isothermal titration calorimetry was performed on a MicroCal PEAQ-ITC Automated (Malvern Panalytical) in PBS at 25 °C with stirring at 750 rpm, 10 µcal/s reference power, and high feedback. The cell was loaded with protein and the syringe with ligand at the indicated concentrations. The titration protocol included an initial delay of 60 s and a first injection of 0.4 µL over 0.8 s, followed by 18 injections of 2 µL over 4 s with a spacing of 150 s. Thermograms were integrated with NITPIC v2.1.5^27^, and isotherms were fitted with SEDPHAT v15.2b^28^ using a single-binding-site hetero-association model.

### Ligand hydrophobicity calculation

The hydrophobicity of canonical peptides was calculated as the sum of individual residue contributions using the Wimley-White interfacial hydrophobicity scale, which for a residue X denotes the free energy of transfer of the AcWLXLL peptide from a 2-oleoyl-1-palmitoyl-*sn*-glycero-3-phosphocholine bilayer interface to water at pH = 8^29^.

### Reanalysis of nutrient utilization assay

The utilization of a peptide attributed to a single *dppA* gene was calculated from the raw data published as “Dataset S3. Raw Data for Data Analysis”^7^ as the difference between the respiratory activity of the *dppA1–5* deletion mutant complemented with that gene and the respiratory activity of the *dppA1–5* deletion mutant.

### Protein structure prediction

The protein structures of DppA1–5 (UniProt IDs see above) were predicted using the ColabFold implementation of AlphaFold2^18^ with ‘num_relax = 5’ and ‘template_mode = pdb100’. The relaxed output structures for each protein were inspected for discrepancies, and none were found. Therefore, the top-ranked structure for each protein was used henceforth and imported into Maestro 13.4 (Schrodinger). The structures were then aligned and superimposed to calculate RMSDs of backbone atoms compared to DppA1.

## Supplementary Information

Below is the link to the electronic supplementary material.

Supplementary Material 1

## Data Availability

Data is provided within the manuscript or supplementary information files. The thermal shifts of DppA1–5 for the entire library of 281 peptide ligands is available in the Supplementary information.
