# Supplementary material for: The solute-binding proteins DppA1–5 of Pseudomonas aeruginosa have distinct substrate profiles
Source: Sci Rep. 2025 Dec 23;15:44346. doi: 10.1038/s41598-025-27865-2 (PMC12728205; doi:10.1038/s41598-025-27865-2)
Supplement: Supplementary file 1 — Supplementary Material 1 [file 41598_2025_27865_MOESM1_ESM.pdf]

Supplementary Information for

The solute-binding proteins DppA1–5 of *Pseudomonas aeruginosa* have distinct substrate profiles

Konstantin Plöchl[1,2,3] & Thomas Böttcher[1,2,*]

[1]Faculty of Chemistry, Institute of Biological Chemistry, University of Vienna, Vienna, Austria

[2]Centre for Microbiology and Environmental Systems Science, Department of Microbiology and Ecosystems Science, University of Vienna, Vienna, Austria

[3]Vienna Doctoral School in Chemistry, University of Vienna, Vienna, Austria

*Corresponding author. E-Mail: thomas.boettcher@univie.ac.at

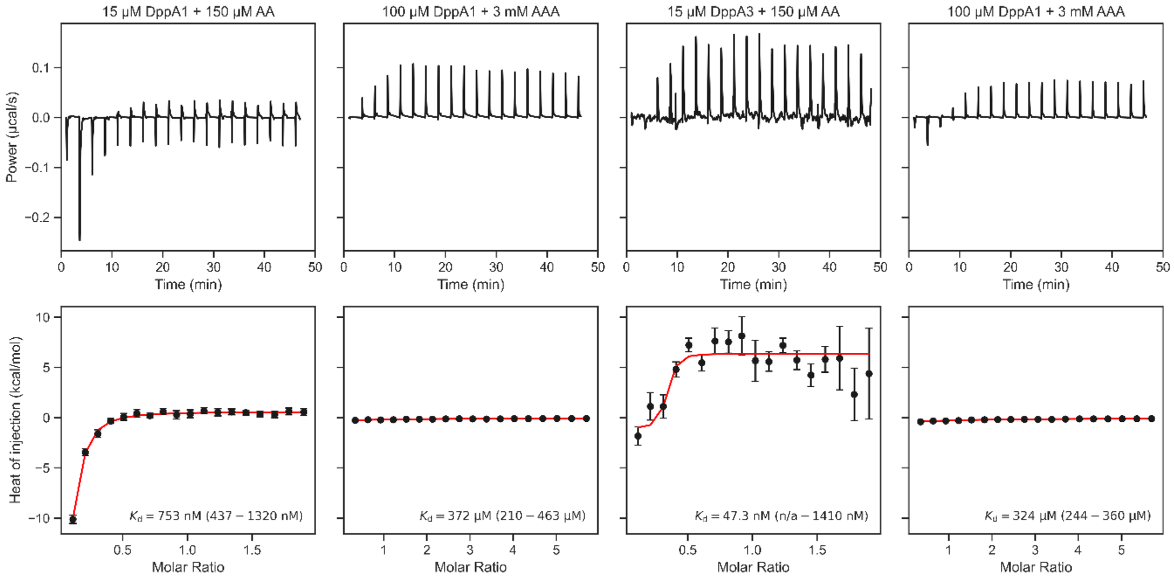

Supplementary Fig. S1. Isothermal titration calorimetry of DppA1 and DppA3 with Ala-Ala and Ala-Ala-Ala. Baseline-corrected thermograms (top) and isotherms with estimated integral errors (bottom). Red lines show fit to single-binding-site model. Dissociation constants are reported with 1σ confidence intervals. For titrations with Ala-Ala-Ala, a control titration of 3 mM Ala-Ala-Ala into buffer was subtracted to exclude dilution effects due to the high injectant concentration. n/a, not available because calculation did not converge.

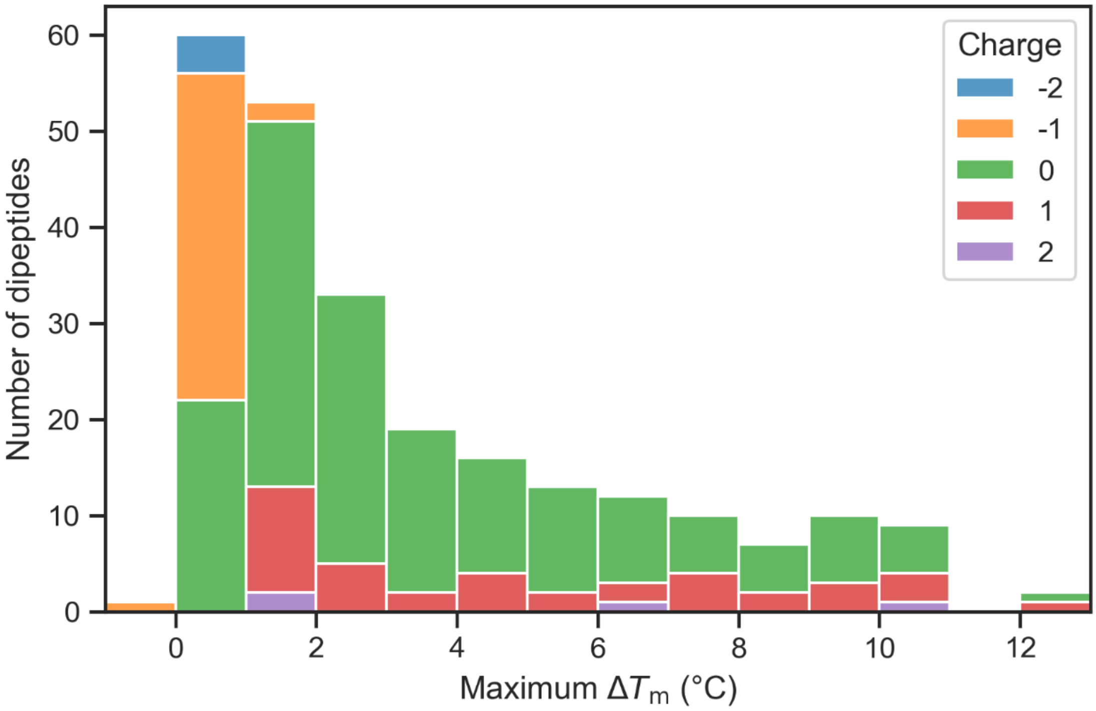

Supplementary Fig. S2. Maximum ΔT_m of canonical dipeptides. For each dipeptide, the maximum thermal shift among DppA1–5 is shown. Ligands are colored by charge at physiological pH.

Supplementary Table S1. Differential Scanning Fluorimetry of *P. aeruginosa* DppA1–5. Thermal shifts in °C of DppA1–5 for the complete library of 281 peptide ligands. Hyp, 4-hydroxyproline.

Substance	Plate	Well	DppA1	DppA2	DppA3	DppA4	DppA5
**AA**	PM6	A3	9.52	2.07	8.11	–2.33	–0.64
**AR**	PM6	A4	12.54	2.06	2.43	–1.92	–0.62
**AN**	PM6	A5	8.97	1.53	1.88	–2.18	–0.48
**AE**	PM6	A6	0.84	0.76	–0.22	–1.94	–0.81
**AG**	PM6	A7	3.52	2.10	1.60	–2.12	–0.82
**AH**	PM6	A8	12.84	1.82	0.91	–1.50	–0.34
**AL**	PM6	A9	5.05	2.08	1.46	–2.52	–0.56
**AK**	PM6	A10	10.32	2.59	0.49	–2.15	–0.40
**AF**	PM6	A11	5.37	2.08	0.68	–2.21	–0.68
**AP**	PM6	A12	1.07	0.99	7.71	–1.94	–1.30
**AS**	PM6	B1	9.81	2.25	3.43	–2.09	–0.23
**AT**	PM6	B2	6.30	2.44	2.52	–1.91	–0.32
**AW**	PM6	B3	1.80	1.31	0.34	–1.84	–0.37
**AY**	PM6	B4	2.25	1.53	0.93	–1.30	–0.40
**RA**	PM6	B5	1.17	1.17	2.65	–1.10	–0.36
**RR**	PM6	B6	1.48	1.28	0.45	–0.86	–0.45
**RD**	PM6	B7	–0.11	0.59	–0.19	–0.53	–0.22
**RQ**	PM6	B8	1.55	1.34	–0.04	–0.79	–0.35
**RE**	PM6	B9	0.08	0.40	–0.02	–0.98	–0.21
**RI**	PM6	B10	1.65	1.24	0.58	–1.21	–0.13
**RL**	PM6	B11	2.36	1.69	0.67	–1.00	–0.15
**RK**	PM6	B12	1.40	1.29	0.18	–1.69	–0.43
**RM**	PM6	C1	1.59	1.10	0.18	–0.99	–0.07
**RF**	PM6	C2	2.13	1.48	0.41	–0.32	–0.24
**RS**	PM6	C3	1.07	1.03	1.29	–0.76	–0.25
**RW**	PM6	C4	1.72	1.16	0.35	–0.18	–0.24
**RY**	PM6	C5	1.51	1.07	0.49	0.07	–0.37
**RV**	PM6	C6	1.48	1.01	2.19	–0.22	–0.39
**NE**	PM6	C7	–0.16	0.46	–0.25	–0.64	–0.26
**NV**	PM6	C8	1.96	1.64	0.59	–0.48	–0.20
**DD**	PM6	C9	–0.14	0.51	–0.17	–0.15	–0.22

**DE**	PM6	C10	–0.10	0.90	–0.10	–1.14	–0.18
**DL**	PM6	C11	0.26	0.60	0.04	–1.14	–0.09
**DK**	PM6	C12	0.14	0.87	0.02	–1.67	–0.31
**DF**	PM6	D1	0.25	0.47	–0.01	–1.01	–0.18
**DW**	PM6	D2	0.10	0.77	0.00	–0.60	–0.16
**DV**	PM6	D3	0.05	0.65	0.04	–0.53	–0.20
**CG**	PM6	D4	–0.05	0.48	–0.09	–0.18	–0.45
**QQ**	PM6	D5	6.85	2.21	–0.17	–0.26	–0.23
**QG**	PM6	D6	1.23	0.95	–0.08	–0.26	–0.41
**ED**	PM6	D7	–0.30	0.27	–0.31	–0.40	–0.34
**EE**	PM6	D8	–0.26	0.87	–0.26	–0.43	–0.33
**EG**	PM6	D9	–0.15	0.73	–0.13	–0.43	–0.19
**ES**	PM6	D10	0.48	0.76	0.03	–0.05	–0.05
**EW**	PM6	D11	0.12	1.02	0.07	–0.49	–0.11
**EY**	PM6	D12	0.15	1.02	0.11	–1.62	–0.27
**EV**	PM6	E1	0.38	0.45	0.33	–0.67	–0.21
**GA**	PM6	E2	3.66	2.12	0.99	–0.38	–0.08
**GR**	PM6	E3	5.52	1.69	0.44	–0.25	–0.27
**GC**	PM6	E4	4.02	2.48	0.42	–0.20	–0.01
**GG**	PM6	E5	0.65	0.70	–0.21	–0.38	–0.14
**GH**	PM6	E6	6.33	2.21	–0.01	–0.24	–0.35
**GL**	PM6	E7	2.12	1.56	0.05	–0.32	–0.36
**GK**	PM6	E8	1.82	1.86	–0.24	–0.37	–0.35
**GM**	PM6	E9	3.63	2.04	0.02	–0.23	–0.05
**GF**	PM6	E10	2.49	1.87	0.46	–0.19	–0.14
**GP**	PM6	E11	0.35	0.77	1.36	–0.71	–0.09
**GS**	PM6	E12	4.28	2.41	0.27	–1.52	–0.18
**GT**	PM6	F1	1.49	1.44	0.07	–0.78	–0.36
**GW**	PM6	F2	1.09	0.78	0.19	–0.34	–0.18
**GY**	PM6	F3	1.15	0.83	0.59	–0.28	–0.40
**GV**	PM6	F4	2.05	1.69	0.62	–0.26	–0.23
**HD**	PM6	F5	–0.19	0.44	–0.25	–0.19	–0.13
**HG**	PM6	F6	0.19	0.34	–0.28	–0.31	–0.22
**HL**	PM6	F7	1.66	1.26	–0.15	–0.21	–0.26
**HK**	PM6	F8	3.16	2.39	–0.21	–0.18	–0.21

**HM**	PM6	F9	3.56	2.46	–0.12	–0.05	–0.06
**HP**	PM6	F10	0.16	0.82	0.68	–0.23	–0.12
**HS**	PM6	F11	3.79	2.05	0.18	–0.31	–0.16
**HW**	PM6	F12	1.25	1.03	0.27	–0.40	–0.22
**HY**	PM6	G1	1.32	0.92	0.30	–0.65	–0.66
**HV**	PM6	G2	2.48	1.99	0.78	–0.31	–0.29
**IA**	PM6	G3	4.89	2.12	6.35	–0.67	–0.28
**IR**	PM6	G4	9.09	2.47	1.61	–0.33	–0.33
**IQ**	PM6	G5	9.35	2.41	0.54	–0.28	–0.24
**IG**	PM6	G6	1.28	1.05	0.98	–0.37	–0.43
**IH**	PM6	G7	8.71	2.48	0.96	–0.14	–0.24
**II**	PM6	G8	1.93	1.65	1.56	–0.34	–0.31
**IM**	PM6	G9	5.36	1.97	0.73	–0.50	–0.19
**IF**	PM6	G10	3.08	2.14	1.33	–0.61	–0.22
**IP**	PM6	G11	0.96	0.91	7.03	–1.00	–0.25
**IS**	PM6	G12	6.26	1.96	2.32	–2.07	–0.64
**IW**	PM6	H1	1.57	1.35	1.04	–2.05	–1.50
**IY**	PM6	H2	1.67	1.18	2.00	–0.88	–0.58
**IV**	PM6	H3	3.87	2.13	4.80	–0.44	–0.51
**LA**	PM6	H4	4.19	2.13	2.15	–0.76	–0.35
**LR**	PM6	H5	9.85	2.21	0.85	0.00	–0.27
**LD**	PM6	H6	–0.21	0.40	–0.46	–0.23	–0.29
**LE**	PM6	H7	0.02	0.39	–0.34	–0.39	–0.53
**LG**	PM6	H8	1.07	1.10	0.08	–0.24	–0.30
**LI**	PM6	H9	3.07	2.34	0.97	–0.15	–0.18
**LL**	PM6	H10	2.27	2.09	0.88	–0.09	–0.39
**LM**	PM6	H11	5.54	2.52	0.21	–0.61	–0.51
**LF**	PM6	H12	2.57	1.78	0.37	–2.32	–1.13
**LS**	PM7	A3	5.85	1.74	0.80	–1.95	–0.68
**LW**	PM7	A4	1.37	0.98	0.36	–2.12	–0.62
**LV**	PM7	A5	4.22	2.15	2.64	–2.30	–0.56
**KA**	PM7	A6	9.04	2.37	1.86	–1.84	–0.45
**KR**	PM7	A7	10.85	2.05	0.29	–1.85	–0.70
**KE**	PM7	A8	1.17	0.85	–0.29	–2.60	–0.74
**KI**	PM7	A9	4.30	2.11	0.18	–2.00	–1.11

**KL**	PM7	A10	5.02	1.95	0.30	–1.69	–1.04
**KK**	PM7	A11	6.60	2.42	–0.11	–2.10	–1.82
**KF**	PM7	A12	7.11	1.76	–0.06	–2.76	–1.84
**KP**	PM7	B1	1.36	1.13	1.23	–1.16	–0.90
**KS**	PM7	B2	10.54	1.87	0.52	–0.70	–0.48
**KT**	PM7	B3	6.86	1.82	0.23	–0.91	–0.33
**KW**	PM7	B4	2.90	2.11	0.17	–0.13	–0.48
**KY**	PM7	B5	3.38	2.14	0.29	–0.69	–0.56
**KV**	PM7	B6	7.20	2.21	1.43	–0.71	–0.41
**MR**	PM7	B7	7.95	2.05	2.47	–1.18	–0.26
**MD**	PM7	B8	–0.02	0.36	–0.29	–1.34	–0.30
**MQ**	PM7	B9	8.34	2.33	0.53	–1.20	–0.45
**ME**	PM7	B10	0.09	0.91	–0.15	–1.25	–0.64
**MG**	PM7	B11	1.68	1.04	1.55	–1.52	–0.47
**MH**	PM7	B12	9.34	2.10	0.93	–1.25	–0.67
**MI**	PM7	C1	2.52	2.00	1.14	–0.41	–0.51
**ML**	PM7	C2	2.85	1.79	1.13	–0.28	–0.40
**MK**	PM7	C3	4.49	1.66	0.02	–0.36	–0.37
**MM**	PM7	C4	4.91	2.18	–0.02	–0.41	–0.44
**MF**	PM7	C5	2.96	2.02	0.29	–0.49	–0.49
**MP**	PM7	C6	0.27	0.47	6.28	–0.55	–0.27
**MW**	PM7	C7	1.34	0.89	0.11	–0.49	–0.50
**MV**	PM7	C8	3.69	2.23	4.90	–0.31	–0.32
**FA**	PM7	C9	2.23	1.69	0.43	–0.74	–0.34
**FG**	PM7	C10	0.35	0.49	–0.10	–0.86	–0.38
**FI**	PM7	C11	2.17	1.58	0.36	–0.34	–0.32
**FF**	PM7	C12	0.06	–0.10	–0.10	–1.72	–0.91
**FP**	PM7	D1	0.29	0.27	0.23	–0.61	–0.46
**FS**	PM7	D2	2.34	1.52	–0.12	–0.26	–0.35
**FW**	PM7	D3	0.41	–0.25	–0.31	–0.85	–0.84
**PA**	PM7	D4	5.31	1.91	1.72	–0.14	–0.13
**PD**	PM7	D5	–0.20	0.67	–0.29	–0.26	–0.15
**PQ**	PM7	D6	4.14	2.13	–0.28	–0.25	–0.09
**PG**	PM7	D7	0.94	0.73	–0.19	–0.29	–0.22
**Pro-Hyp**	PM7	D8	–0.21	0.38	–0.22	–0.28	–0.25

**PL**	PM7	D9	2.94	2.11	–0.02	–0.11	–0.19
**PF**	PM7	D10	2.89	2.22	0.11	–0.01	–0.17
**PP**	PM7	D11	0.28	0.52	1.67	0.00	–0.19
**PY**	PM7	D12	0.98	0.21	0.23	–0.08	–0.66
**SA**	PM7	E1	5.72	2.06	3.36	–0.62	–0.25
**SG**	PM7	E2	1.78	1.43	0.54	–0.14	–0.24
**SH**	PM7	E3	10.02	2.15	0.29	0.47	–0.19
**SL**	PM7	E4	3.69	2.10	0.30	–0.24	–0.17
**SM**	PM7	E5	6.34	2.25	–0.29	–0.27	–0.26
**SF**	PM7	E6	3.75	2.07	–0.02	–0.24	–0.31
**SP**	PM7	E7	0.27	0.25	4.45	–0.34	–0.28
**SS**	PM7	E8	6.33	2.45	1.29	–0.30	–0.26
**SY**	PM7	E9	1.81	1.51	0.58	–0.11	–0.35
**SV**	PM7	E10	5.21	2.21	2.58	–0.15	–0.29
**TA**	PM7	E11	2.38	1.79	2.65	–0.02	–0.28
**TR**	PM7	E12	4.63	2.16	0.86	–0.25	–0.57
**TE**	PM7	F1	0.09	0.46	–0.34	–1.27	–0.36
**TG**	PM7	F2	0.49	0.44	0.12	–0.27	–0.31
**TL**	PM7	F3	1.92	1.54	–0.01	–0.28	–0.16
**TM**	PM7	F4	2.77	2.20	–0.23	–0.18	–0.20
**TP**	PM7	F5	0.34	0.45	5.53	–0.11	–0.20
**WA**	PM7	F6	1.62	1.70	0.49	–0.34	–0.42
**WR**	PM7	F7	1.37	1.13	–0.11	–0.26	–0.22
**WD**	PM7	F8	–0.19	0.37	–0.41	–0.35	–0.37
**WE**	PM7	F9	–0.06	0.53	–0.35	–0.25	–0.30
**WG**	PM7	F10	0.36	0.56	–0.33	–0.46	–0.58
**WL**	PM7	F11	2.34	1.51	–0.02	–0.31	–0.40
**WK**	PM7	F12	1.25	0.82	0.00	–1.12	–0.81
**WF**	PM7	G1	2.28	1.46	0.17	–2.16	–0.73
**WS**	PM7	G2	2.04	1.38	–0.14	–0.16	–0.41
**WW**	PM7	G3	1.07	0.55	–0.44	–0.37	–0.40
**WY**	PM7	G4	2.32	1.58	0.33	–0.33	–0.41
**YA**	PM7	G5	0.79	0.87	–0.11	–0.25	–0.36
**YQ**	PM7	G6	0.67	0.43	–0.48	–0.33	–0.54
**YE**	PM7	G7	–0.19	0.65	–0.44	–0.23	–0.36

**YG**	PM7	G8	–0.12	0.48	–0.45	–0.34	–0.63
**YH**	PM7	G9	1.81	1.36	0.08	–0.09	–0.35
**YL**	PM7	G10	1.46	0.81	–0.20	–0.36	–0.42
**YK**	PM7	G11	1.77	1.26	–0.23	–0.36	–0.56
**YF**	PM7	G12	1.18	0.18	–0.19	–1.67	–1.24
**YW**	PM7	H1	0.85	0.26	–0.55	–2.21	–1.33
**YY**	PM7	H2	1.39	0.69	–0.32	–0.21	–0.78
**VR**	PM7	H3	10.13	1.61	1.71	–0.12	–0.35
**VN**	PM7	H4	7.00	1.60	1.91	–0.62	–0.63
**VD**	PM7	H5	–0.03	0.26	–0.62	–0.29	–0.35
**VG**	PM7	H6	1.92	1.38	2.12	–0.27	–0.49
**VH**	PM7	H7	10.85	1.98	1.00	0.06	–0.70
**VI**	PM7	H8	3.69	2.01	2.17	–0.31	–0.41
**VL**	PM7	H9	3.96	2.16	1.93	–0.57	–0.50
**VY**	PM7	H10	1.79	1.12	1.84	–1.62	–0.83
**VV**	PM7	H11	6.10	2.07	9.95	–1.59	–1.15
**AD**	PM8	A3	0.13	0.11	–0.49	–2.39	–1.66
**AQ**	PM8	A4	10.67	1.89	0.27	–3.11	–1.54
**AI**	PM8	A5	4.22	2.11	1.04	–2.25	–1.08
**AM**	PM8	A6	9.41	1.98	–0.03	–2.06	–1.41
**AV**	PM8	A7	7.47	2.05	6.30	–1.56	–1.34
**DA**	PM8	A8	0.05	0.33	–0.33	–1.91	–0.90
**DQ**	PM8	A9	0.28	0.12	–0.53	–2.42	–1.27
**DG**	PM8	A10	0.07	0.03	–0.34	–2.27	–1.49
**EA**	PM8	A11	0.44	0.33	0.17	–2.61	–1.27
**GN**	PM8	A12	3.12	1.09	–0.34	–3.05	–1.88
**GD**	PM8	B1	0.05	–0.19	–0.36	–1.20	–0.94
**GI**	PM8	B2	1.58	0.99	–0.05	–1.44	–0.60
**HA**	PM8	B3	2.42	1.82	0.43	–0.88	–0.45
**HE**	PM8	B4	0.04	0.20	–0.21	–0.36	–0.44
**HH**	PM8	B5	4.63	2.07	–0.13	–0.50	–0.40
**IN**	PM8	B6	5.18	2.07	1.10	–1.23	–0.49
**IL**	PM8	B7	2.95	1.89	1.38	–0.94	–0.46
**LN**	PM8	B8	5.32	0.43	0.63	–2.13	–0.47
**LH**	PM8	B9	9.42	1.94	0.41	0.40	–0.41

**LP**	PM8	B10	0.31	0.26	1.70	–1.59	–0.44
**LY**	PM8	B11	1.44	0.85	0.98	–1.48	–0.50
**KD**	PM8	B12	0.29	0.24	–0.27	–2.23	–0.99
**KG**	PM8	C1	4.91	2.15	0.10	–0.67	–0.43
**KM**	PM8	C2	8.71	2.20	–0.25	–0.24	–0.48
**MT**	PM8	C3	2.23	1.62	1.50	–0.58	–0.37
**MY**	PM8	C4	1.56	0.98	0.63	–0.35	–0.48
**FD**	PM8	C5	–0.18	0.01	–0.41	–0.74	–0.49
**FE**	PM8	C6	–0.17	–0.22	–0.37	–0.59	–0.56
**QE**	PM8	C7	–0.04	0.08	–0.39	–1.05	–0.46
**FM**	PM8	C8	3.13	1.79	–0.26	–0.59	–0.57
**FY**	PM8	C9	1.35	0.73	0.34	–0.62	–0.54
**FV**	PM8	C10	2.99	1.88	0.35	–1.01	–0.30
**PR**	PM8	C11	8.67	1.94	0.72	–0.61	–0.21
**PN**	PM8	C12	3.15	1.88	0.01	–1.24	–0.35
**PE**	PM8	D1	0.05	0.29	–0.18	–0.16	–0.31
**PI**	PM8	D2	2.27	1.71	0.08	0.03	–0.19
**PK**	PM8	D3	6.99	2.07	–0.04	–0.05	–0.09
**PS**	PM8	D4	4.78	2.26	0.59	–0.19	–0.25
**PW**	PM8	D5	0.36	0.12	–0.21	–0.26	–0.27
**PV**	PM8	D6	3.41	2.02	1.34	–0.18	–0.18
**SN**	PM8	D7	6.63	2.05	0.81	–0.24	–0.22
**SD**	PM8	D8	–0.17	0.21	–0.39	–0.40	–0.25
**SQ**	PM8	D9	8.36	1.84	–0.10	–0.43	–0.27
**SE**	PM8	D10	0.37	0.33	–0.18	–0.46	–0.21
**TD**	PM8	D11	0.04	0.28	–0.18	–1.08	–0.43
**TQ**	PM8	D12	3.72	2.20	–0.10	–1.49	–0.82
**TF**	PM8	E1	1.98	1.26	0.24	–0.30	–0.33
**TS**	PM8	E2	0.94	0.83	0.18	–0.35	–0.36
**WV**	PM8	E3	1.82	1.20	0.69	–0.25	–0.35
**YI**	PM8	E4	1.23	0.78	–0.07	–0.24	–0.44
**YV**	PM8	E5	1.38	1.21	–0.01	–0.34	–0.42
**VA**	PM8	E6	6.98	2.00	10.11	–0.36	–0.43
**VQ**	PM8	E7	8.66	1.95	0.82	–0.40	–0.40
**VE**	PM8	E8	0.23	0.15	–0.33	–0.54	–0.38

**VK**	PM8	E9	7.84	2.12	0.54	–0.27	–0.40
**VM**	PM8	E10	7.40	1.94	0.70	–0.62	–0.37
**VF**	PM8	E11	3.78	1.87	1.41	–1.03	–0.20
**VP**	PM8	E12	0.58	0.30	10.27	–1.77	–0.97
**VS**	PM8	F1	7.49	1.74	5.52	–1.03	–0.52
**β-Ala-Ala**	PM8	F2	0.04	0.31	–0.34	–0.52	–0.51
**β-Ala-Gly**	PM8	F3	–0.20	0.17	–0.43	–0.60	–0.57
**β-Ala-His**	PM8	F4	0.47	0.29	–0.15	–0.22	–0.40
**Met-β-Ala**	PM8	F5	–0.19	0.10	–0.25	–0.22	–0.25
**β-Ala-Phe**	PM8	F6	0.27	–0.05	–0.37	–0.43	–0.47
**D-Ala-D-Ala**	PM8	F7	–0.29	0.04	–0.45	–0.45	–0.46
**D-Ala-Gly**	PM8	F8	–0.27	0.08	–0.43	–0.51	–0.37
**D-Ala-Leu**	PM8	F9	0.12	0.08	–0.32	–0.62	–0.26
**D-Leu-D-Leu**	PM8	F10	–0.15	–0.01	–0.37	–1.07	–0.42
**D-Leu-Gly**	PM8	F11	–0.07	0.19	–0.27	–1.34	–0.35
**D-Leu-Tyr**	PM8	F12	0.45	0.02	–0.09	–2.01	–1.37
**γ-Glu-Gly**	PM8	G1	–0.08	0.02	–0.47	–1.67	–0.97
**γ-D-Glu-Gly**	PM8	G2	–0.09	0.48	–0.46	–0.95	–0.57
**Gly-D-Ala**	PM8	G3	–0.10	0.17	–0.36	–0.54	–0.76
**Gly-D-Asp**	PM8	G4	–0.18	0.29	–0.33	–0.41	–0.37
**Gly-D-Ser**	PM8	G5	–0.13	0.26	–0.32	–0.36	–0.44
**Gly-D-Thr**	PM8	G6	–0.25	–0.02	–0.40	–0.45	–0.43
**Gly-D-Val**	PM8	G7	–0.31	0.06	–0.33	–0.34	–0.20
**Leu-β-Ala**	PM8	G8	–0.16	–0.01	–0.35	–0.39	–0.35
**Leu-D-Leu**	PM8	G9	–0.16	0.04	–0.38	–0.63	–0.39
**Phe-β-Ala**	PM8	G10	0.05	0.19	–0.27	–0.52	–0.34
**Ala-Ala-Ala**	PM8	G11	0.52	10.61	1.09	5.88	–0.45
**D-Ala-Gly-Gly**	PM8	G12	–0.05	3.47	–0.47	–1.89	–1.08
**Gly-Gly-Ala**	PM8	H1	0.05	9.58	–0.64	0.24	–1.38
**Gly-Gly-D-Leu**	PM8	H2	–0.09	0.16	–0.61	–1.37	–0.66
**Gly-Gly-Gly**	PM8	H3	–0.10	6.59	–0.39	–0.86	–0.47
**Gly-Gly-Ile**	PM8	H4	–0.05	6.62	–0.31	3.22	–0.59
**Gly-Gly-Leu**	PM8	H5	–0.11	8.22	–0.32	2.66	–0.35
**Gly-Gly-Phe**	PM8	H6	–0.13	10.30	–0.39	0.77	–0.36
**Val-Tyr-Val**	PM8	H7	–0.17	6.12	–0.46	5.40	–0.41

**Gly-Phe-Phe**	PM8	H8	0.11	7.83	–0.44	3.68	–0.14
**Leu-Gly-Gly**	PM8	H9	–0.21	5.25	–0.48	0.07	–0.30
**Leu-Leu-Leu**	PM8	H10	0.15	7.13	–0.38	8.76	–0.39
**Phe-Gly-Gly**	PM8	H11	–0.01	5.07	–0.43	–1.69	–0.59
**Tyr-Gly-Gly**	PM8	H12	–0.01	3.20	–0.54	–2.30	–1.54
